# Comparative Study of Hyperbaric Bupivacaine Versus Levobupivacaine for Rapid Sequence Spinal Anesthesia in Category 1 and 2 Cesarean Sections

**DOI:** 10.7759/cureus.110283

**Published:** 2026-06-05

**Authors:** Puja Choubey, Tanushree Srivastava, Abhishek Srivastava

**Affiliations:** 1 Department of Anaesthesiology, Integral Institute of Medical Sciences and Research, Lucknow, IND

**Keywords:** category 1&2 cesarean section, hemodynamic stability, hyperbaric bupivacaine, levobupivacaine, motor block characteristics, obstetrics anesthesia, rapid sequence spinal anesthesia

## Abstract

Introduction

Spinal anesthesia (SA) is widely preferred for cesarean sections (CS) due to its association with decreased mortality compared to general anesthesia. This preference applies to urgent clinical situations where prompt delivery is required because of maternal or fetal concerns. To minimize time constraints in these urgent situations, “rapid sequence SA (RSSA),” which involves executing only essential steps and limiting administration attempts, has emerged as a safer and faster alternative. Among the local anesthetics used for spinal anesthesia, hyperbaric bupivacaine and levobupivacaine are widely utilized agents.

Methods

This prospective, randomized, double-blind clinical study evaluated the effects of a fixed 12.5 mg dose of hyperbaric levobupivacaine (Group B) compared with hyperbaric bupivacaine (Group A) for RSSA in 100 pregnant women undergoing Category 1 or 2 CS. The study protocol, registered with Clinical Trials Registry-India (CTRI) (CTRI/2023/04/051557), omitted opioid use and restricted local anesthetic administration attempts.

Results

The results indicated that sensory block parameters (onset time, maximum level T4, and regression time) between the groups were comparable, with no statistically significant difference. However, the mean time to achieve motor block onset was significantly faster in Group A (bupivacaine) (172.4 s vs. 214.64 s, p = 0.008). Similarly, motor regression time was significantly longer in Group A. Although overall hemodynamic variables were similar between the groups, hypotension and bradycardia occurred more frequently in the bupivacaine group; however, this difference did not reach statistical significance (p ≥ 0.05).

Conclusion

In conclusion, both fixed doses of bupivacaine and levobupivacaine are effective and reliable for RSSA in Category 1 and 2 CS. Hyperbaric bupivacaine demonstrated more favorable motor block properties, whereas levobupivacaine was linked to improved hemodynamic stability.

## Introduction

Spinal anesthesia is a widely preferred technique for cesarean section (CS) due to its association with decreased anesthesia-related mortality compared to general anesthesia [1]. It provides effective analgesia, anesthesia, and motor block, with its effect being dependent on the drug's volume, concentration, and dose [2]. Historically, 0.5% hyperbaric bupivacaine has been the most common choice for spinal anesthesia in lower segment CS (LSCS), although its use is not without risks [3].

Levobupivacaine, the S(-)-isomer of bupivacaine, received approval from the U.S. Food and Drug Administration in 1997 and is used as an alternative local anesthetic. Although certain studies report comparable clinical efficacy to bupivacaine, others indicate that levobupivacaine may be associated with a lower incidence of adverse effects, including hypotension, bradycardia, and nausea [4,5].

CS are categorized by urgency [6]: Category 1: an urgent situation involving an immediate risk to the life of the mother or fetus; Category 2: evidence of maternal or fetal compromise without an immediate threat to life; Category 3: early delivery is necessary, although there is no current maternal or fetal compromise; Category 4: the timing of delivery is planned at the convenience of the woman and the maternity care team.

To reduce delays, especially in Category 1 CS, rapid sequence spinal anesthesia (RSSA) has been introduced as a novel technique [7]. It was developed as a safer substitute for emergency general anesthesia, which is associated with potentially fatal complications [6], and as a quicker alternative to traditional spinal anesthesia, which can be time-consuming. RSSA integrates elements of both techniques, emphasizing the rapid establishment of a subarachnoid block by carrying out only the critical steps. This approach shortens the allowable preparation time, restricts the number of attempts, and mandates abandonment of the block if delays arise, analogous to rapid sequence induction [7]. While common in emergency obstetric anesthesia, the rapidity, dose, and effects of RSSA are often undocumented. This study aims to compare fixed doses of hyperbaric levobupivacaine and hyperbaric bupivacaine in Category 1 and 2 cesarean deliveries conducted under RSSA, focusing on sensory and motor block characteristics as well as hemodynamic stability.

## Materials and methods

The study protocol received approval from the Ethics Committee and was enrolled in the Clinical Trials Registry-India (CTRI) with registration number CTRI/2023/04/051557, registered on April 12, 2023. A total of 100 patients scheduled for Category 1 and 2 CS were selected for the study at Maharani Laxmi Bai (MLB) Medical College and Hospital, Jhansi, India. This was a double-blinded, prospective, randomized, clinical trial. Participants were randomly allocated to two equal-sized intervention groups (Group A and Group B) using an open random number list. The study was conducted from April 2023 to September 2023. Following the acquisition of written informed consent, patients were randomly assigned to two groups of 50 each using a computer-generated randomization table. Group A was administered 12.5 mg of hyperbaric bupivacaine intrathecally, while Group B received 12.5 mg of hyperbaric levobupivacaine intrathecally. Out of the total enrolled patients, the number of patients converted to general anesthesia due to inadequate block height and subsequently excluded from the study was two in Group A and three in Group B.

Inclusion criteria

Inclusion criteria include pregnant women of gestational age > 37 weeks, aged between 20 and 40 years, and posted for Category 1 and 2 CS.

Exclusion criteria

Exclusion criteria include patient refusal, severe hemodynamic instability, contraindications to spinal anesthesia, known allergy to local anesthetics, spinal fusion, musculoskeletal abnormalities, and coagulation disorders.

Components of RSSA

Other staff were deployed for intravenous cannulation and monitoring, and spinal injection was only performed once the cannula was secured [8]. Pre-oxygenation was done during the attempt. A “no-touch” approach was used, where only gloves were worn, and the glove packing served as a sterile surface for the equipment. The skin was disinfected with a single application of 0.5% chlorhexidine solution. The use of opioids was omitted, and a fixed dose of local anesthetic (12.5 mg of hyperbaric bupivacaine or 12.5 mg of hyperbaric levobupivacaine) was administered. Infiltration with local anesthesia was not mandatory. Only a single attempt at spinal injection was made unless an obvious correction allowed for a second. If necessary, surgery was allowed to start when the block reached at least T10 dermatome and was ascending, with preparation for conversion to general anesthesia if required.

Intervention and evaluation

On entering the operating theater, patients were positioned in the left lateral decubitus position. An 18 G intravenous cannula was inserted, and supplemental oxygen was provided via face mask. Prophylactic medications-8 mg of intravenous ondansetron and 50 mg of intravenous ranitidine-were administered, followed by co-loading with 15 mL/kg of normal saline. Baseline vital signs were recorded, and continuous monitoring was established using a multiparameter monitor that included pulse oximetry, electrocardiography, and non-invasive blood pressure measurement.

A 25 G Quincke spinal needle was inserted into the subarachnoid space using a no-touch technique through a midline approach at the L2-L3 or L3-L4 intervertebral level, with correct placement confirmed by continuous free flow of CSF. Following confirmation, the study drug was given intrathecally, after which patients were immediately positioned supine. Throughout the procedure, intravenous fluid therapy was maintained using either normal saline or Ringer's lactate solution. Surgery was permitted to commence once the upper level of sensory block reached at least T10 dermatome and was ascending.

The study medication was prepared by an anesthesiologist responsible for randomization but not involved in any other part of the study. The anesthesiologist who administered the drug also monitored the study parameters. Consequently, both the patients and the observer were blinded to the identity of the study drug.

Parameters studied

For sensory blockade evaluation, parameters assessed included onset time, time to achieve maximum sensory level, highest level attained, time to two-dermatome regression, and time to regression to the T12 dermatome. For the evaluation of the onset of motor blockade, parameters assessed included the time of onset, time to achieve Bromage score 3 or 4, and time to regression. Evaluation of hemodynamic parameters and adverse effects was also conducted.

Definitions of study parameters

The onset of sensory block was defined as the time from intrathecal injection to the initial appearance of analgesia at the T12 dermatome, assessed using the pinprick method. The upper level of sensory block was defined as the highest dermatome at which sensation was lost, measured from the time of drug administration to the attainment of block at that level.

The onset of motor block was defined as the duration from the first signs of weakness to complete loss of motor function, indicated by the patient’s inability to lift their legs. Modified Bromage scale [9]: 0 = no motor block; 1 = motor block of the hip; 2 = motor block of the hip and knee; 3 = motor block of the hip, knee, and foot. Duration for two-segment regression was defined as the time required for the sensory level to recede by two dermatomes from the maximum level achieved.

Statistical analysis

Data were gathered, organized, coded, and analyzed using SPSS® version 29.0 (IBM Corp., Armonk, NY, USA). Continuous variables were expressed as mean ± standard deviation (SD), and categorical variables were presented as percentages. Comparisons of continuous variables were made using unpaired Student’s t-test. A p-value < 0.05 was considered statistically significant.

**Figure 1:**
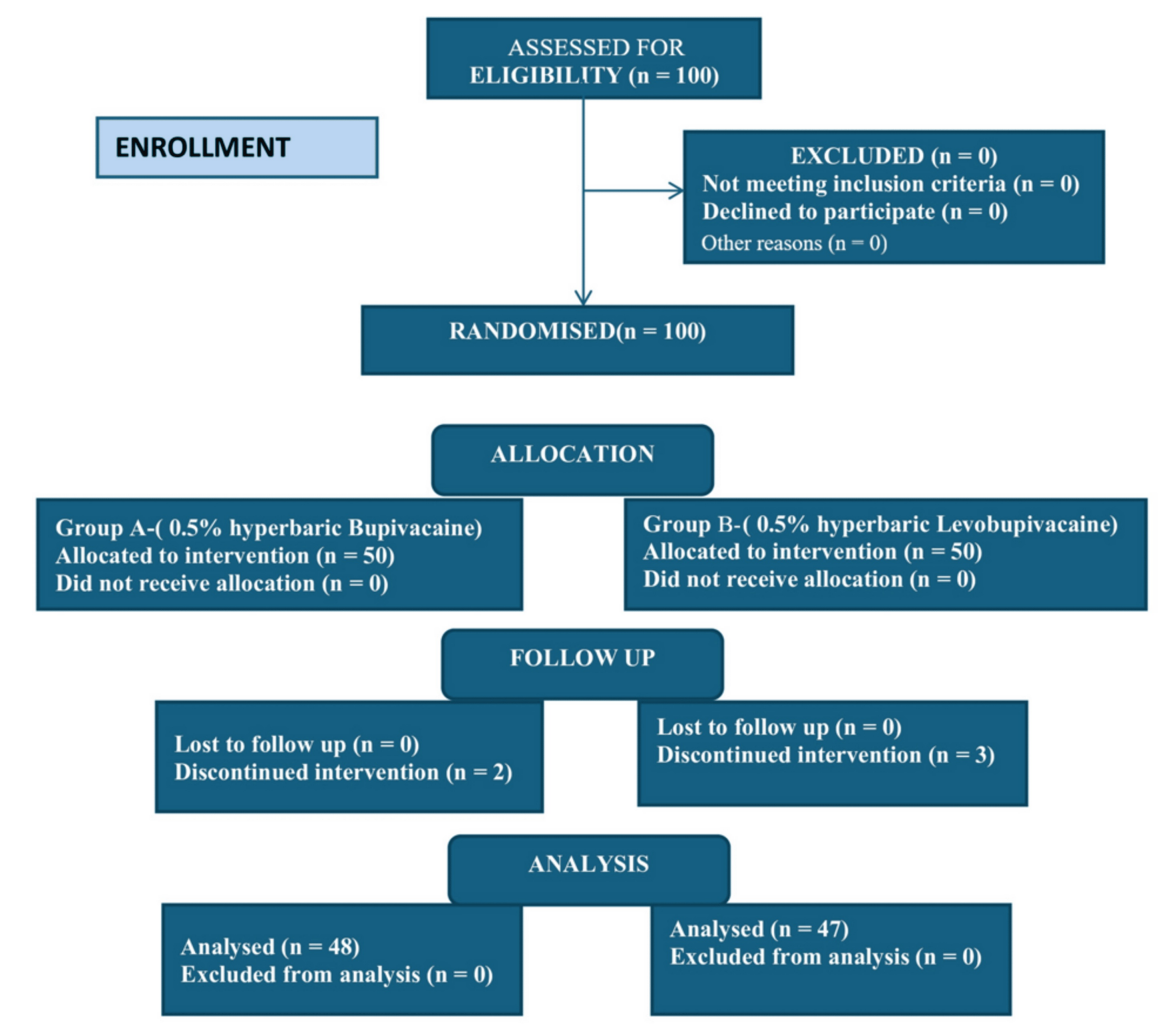
CONSORT Diagram CONSORT: CONsolidated Standards of Reporting Trials

The CONSORT (CONsolidated Standards of Reporting Trials) flow diagram depicting patient enrollment and allocation is shown in Figure 1.

## Results

Demographic characteristics

Demographic parameters were comparable between Group A (bupivacaine) and Group B (levobupivacaine), as shown in Table 1.

**Table 1:** Demographic Profile

	Group A (n = 48)	Group B (n = 47)	p-value
Age (years)	24.3 ± 3.26	23.1 ± 4.28	0.118
Weight (kg)	53.56 ± 5.32	54.42 ± 5.86	0.441
Height (cm)	158 ± 6.45	157 ± 4.84	0.382

Sensory block characteristics

As shown in Table 2, Group A exhibited a slightly faster onset of sensory block and a shorter time to reach the T10 dermatome compared to Group B, though the difference was not statistically significant. The mean onset time of sensory block was 63.80 s in Group A and 64.12 s in Group B (p = 0.8). The two-dermatome regression time was slightly longer in Group B, but this difference was not statistically significant. Overall, all sensory block parameters were comparable between the groups. Both groups achieved a maximum sensory block level of T4, and the time to reach this peak level showed no significant difference. Similarly, the duration for two-segment regression of the sensory block was comparable in both groups, with no statistically significant difference.

**Table 2:** Sensory Block Characteristics

	Group A (n = 48)	Group B (n = 47)	p-value
Time of onset of sensory block (seconds)	63.80 ± 6.78	64.12 ± 5.82	0.8058
Time to reach T10 (seconds)	163.84 ± 7.68	164.44 ± 6.42	0.6808
Time to reach maximum level (seconds)	304.48 ± 68.48	306.48 ± 44.16	0.8365
Maximum level reached	T4	T4	
Time to regression by 2 dermatomes for sensory block (minutes)	110.82 ± 5.84	108.68 ± 6.12	0.084
Regression time to T12 for sensory block (minutes)	130.64 ± 19.436	128.46 ± 18.844	0.549

Motor block characteristics

As presented in Table 3, the mean onset time for motor block was significantly faster in Group A (166.68 s) than in Group B (198.68 s), with a p-value of 0.005, indicating statistical significance. The mean duration for motor block regression was significantly longer in Group A than in Group B, with the difference reaching statistical significance. The time to reach the maximum motor level was comparable in both groups.

**Table 3:** Motor Block Characteristics

	Group A (n = 48)	Group B (n = 47)	p-value
Time of onset of motor block (seconds)	166.68 ± 43.44	198.68 ± 64.80	0.005
Time to reach Bromage 3/4 (seconds)	342.65 ± 56.48	342.46 ± 54.44	0.3911
Time to regression (minutes)	154.84 ± 62.88	128.64 ± 61.12	0.0426

Hemodynamic parameters

Hemodynamic parameters were comparable in both groups, with no statistically significant difference.

Adverse effects

The bupivacaine group experienced a higher incidence of hypotension (12.50%, 6/48 patients) compared to the levobupivacaine group (8.51%, 4/47 patients), though this difference was not statistically significant (p ≥ 0.05). Likewise, bradycardia occurred more frequently in Group A (five patients, 10.41%) than in Group B (four patients, 8.51%), with no statistically significant difference between the groups, as shown in Table 4.

**Table 4:** Side Effects

Complications	Group A	Group B	p-value
Hypotension	6 (12.50%)	4 (8.51%)	0.74
Bradycardia	5 (10.41%)	4 (8.51%)	1.00
Vomiting	6 (12.50%)	7 (14.89%)	0.73
Headache	5 (10.41%)	4 (8.51%)	1.00
Itching	1 (2.08%)	1 (2.12%)	1.00
Backache	6 (12.50%)	4 (8.51%)	0.74
Sedation	1 (2.08%)	2 (4.25%)	0.61
Shivering	7 (14.58%)	8 (17.02%)	0.78

## Discussion

Regional anesthesia plays a crucial role in obstetric anesthesia. The aim of this study was to compare fixed doses of hyperbaric bupivacaine and levobupivacaine for RSSA in Category 1 and 2 cesarean deliveries. The results indicate that RSSA is a fast, effective, and reliable option for Category 1 and 2 CS, offering benefits for both the mother and the baby, and is associated with short preparation and application times, as well as positive neonatal outcomes.

Hemodynamic parameters were comparable in both groups, with no statistically significant differences observed, consistent with the observations of Bekkam et al. [10]. In contrast, Goyal et al. [11] reported a statistically significant variation in systolic blood pressure, while other hemodynamic measures remained similar. Comparable results were also noted by Mandal et al. [12] in their comparison of different bupivacaine doses.

In terms of sensory block, patients in Group A (bupivacaine) demonstrated a slightly faster mean onset compared to Group B (levobupivacaine), but this difference was statistically not significant (63.80 vs. 64.12 s; p = 0.8), consistent with previous studies [4,10,11]. In contrast, Mandal et al. [12] reported a significant difference in the interval from spinal injection to skin incision-a parameter correlating with sensory block onset-likely due to their use of varying doses of the same drug. The maximum sensory block level achieved in both groups was T4, consistent with findings from other studies [13-15]. The mean time to reach the peak sensory level was also comparable between the groups and was not statistically significant, corroborating earlier research [8,13,15]. Additionally, the mean duration for two-segment regression of sensory block was comparable in both groups, with no significant difference, in agreement with the observations of Glaser et al. [14] and Bekkam et al. [10].

Regarding motor block, Group A (bupivacaine) exhibited a significantly faster onset (166.68 s) compared to Group B (levobupivacaine, 198.68 s). Similarly, the duration to regression of motor block was significantly longer in Group A (154.84 s) than in Group B (128.64 s). These results are consistent with previous studies by Glaser et al. [14], Bekkam et al. [10], Luck et al. [4], Fattorini et al. [13], and Choubey et al. [15]. However, the time to achieve Bromage 3/4 motor block was comparable between the groups and showed no statistically significant difference.

Regarding side effects, hypotension was slightly more common in the bupivacaine group (12.51%, 6/48) than in the levobupivacaine group (8.51%, 4/47), although this difference was not statistically significant (p ≥ 0.05). These findings are in line with Luck et al. [4], who reported higher rates of intraoperative hypotension with bupivacaine (42.50%) versus levobupivacaine (17.51%). Similarly, bradycardia occurred more often in Group A (five patients, 10.42%) compared to Group B (four patients, 8.51%), consistent with observations by Luck et al. [4]. Overall, no statistically significant differences were observed in the incidence of side effects between the two groups. This study's limitations included its single-center design and relatively small sample size.

## Conclusions

In conclusion, the results demonstrate that fixed doses of both bupivacaine and levobupivacaine can be successfully employed in RSSA for Category 1 and 2 CS regarding onset, adequacy, level, and duration of block, as well as hemodynamic parameters. This study suggests that hyperbaric bupivacaine provides superior motor block characteristics compared to levobupivacaine. However, levobupivacaine was found to be associated with greater hemodynamic stability.
